# Latest Type of German Tuberculosis Pavilions

**Published:** 1914-11-21

**Authors:** 


### THE NEW TUBERCULOSIS HOSPITAL AT LÜBECK.

### Latest Type of German Tuberculosis Pavilions.

BY A TUBERCULOSIS MEDICAL OFFICER.

My visit to the hospital in Lübeck had for its chief objective the viewing of the new tuberculosis buildings, which have been designed partly by Professor Deycke, to whose methods of tuberculin treatment allusion will later be made. These pavilions were opened in July last. The hospital which serves the ancient city of Lübeck is, like the Eppendorfer Krankenhaus, placed on the outskirts of the town, and covers a considerable area, being planned on similar lines, though naturally on a much smaller scale. The grounds are as yet only partially laid out, and have suffered much at the hands of builders, for a good deal of reconstruction has been going on recently, and the tuberculosis buildings are not the only new ones. The hospital is provided with buildings for dealing with all classes of patients, including infectious diseases. There is an excellent *x*-ray and electrical department, and the bacteriological department has been doing a large amount of work for the tuberculosis division in connection with complement-fixation tests to control the results of the Deycke-Much treatment.

The tuberculosis wards to which attention was specially directed present several points of interest, and as they are so very new the innovations observed must be regarded as the latest word in habitations for pulmonary tuberculosis patients.

Two features spring at once into notice on entering the pavilion, the one is the colouring of the walls, and the other is the fact that the wards open one into another. The walls not only of the corridors, but also of the wards, are painted in most brilliant colours, unrelieved by any designs or dadoes; the doors in these walls are beautifully fitted, quite flush with the walls, and are without panelling. They are painted in equally brilliant hues, but in marked contrast to the colour of the adjoining wall. For example, a bright blue door will be seen in a brilliant yellow wall. A large range of colours is employed in the pavilion, and all are bright tones, giving an impression almost Futurist in character. These details were arranged by Professor Deycke with a view to imparting cheerful contrasts and avoiding any suggestion of monotony in the surroundings of his patients.

The wards themselves are not large, some containing only eight or ten beds; they are well lighted, and the wall colouring certainly makes for cheerfulness. The *Liege-halle* is something more than a verandah: it is commodious, well sheltered, and has a pleasant outlook. Beds can easily be brought in if necessary, but couches and comfortable chairs are most in use. The tuberculosis block is practically independent, and has its own storerooms, kitchens, dispensary, and a laboratory. There is also a clinical room, where special examinations are made on the patients, such as puncture of the thorax, limited *x*-ray work, blood examinations, and pressure estimations. The sterilisation of sputum cups, etc., is carried out in this block.

It is interesting to note that the floors in the corridors are covered with tiles, and not with any of the modern compositions. Inquiries made showed that these tiles were considered very satisfactory, while their wearing properties had proved equal to any demands. The floor of the verandah is paved with wood blocks. The patients wear hospital suits.

